# Gulls as Sources of Environmental Contamination by Colistin-resistant Bacteria

**DOI:** 10.1038/s41598-020-61318-2

**Published:** 2020-03-10

**Authors:** Alan B. Franklin, Andrew M. Ramey, Kevin T. Bentler, Nicole L. Barrett, Loredana M. McCurdy, Christina A. Ahlstrom, Jonas Bonnedahl, Susan A. Shriner, Jeffrey C. Chandler

**Affiliations:** 1 U.S. Department of Agriculture, National Wildlife Research Center, Fort Collins, CO USA; 2 U.S. Geological Survey, Alaska Science Center, Anchorage, AK USA; 3 Department of Clinical and Experimental Medicine, Linköping University, Linköping, Sweden; 4 Department of Infectious Diseases, Region Kalmar County, Kalmar, Sweden

**Keywords:** Ecological epidemiology, Antimicrobial resistance, Bacterial infection

## Abstract

In 2015, the *mcr-1* gene was discovered in *Escherichia coli* in domestic swine in China that conferred resistance to colistin, an antibiotic of last resort used in treating multi-drug resistant bacterial infections in humans. Since then, *mcr-1* was found in other human and animal populations, including wild gulls. Because gulls could disseminate the *mcr-1* gene, we conducted an experiment to assess whether gulls are readily colonized with *mcr-1* positive *E. coli*, their shedding patterns, transmission among conspecifics, and environmental deposition. Shedding of *mcr-1 E. coli* by small gull flocks followed a lognormal curve and gulls shed one strain >10^1^ log10 CFU/g in their feces for 16.4 days, which persisted in the environment for 29.3 days. Because gulls are mobile and can shed antimicrobial-resistant bacteria for extended periods, gulls may facilitate transmission of *mcr-1* positive *E. coli* to humans and livestock through fecal contamination of water, public areas and agricultural operations.

## Introduction

In 2015, a new antimicrobial resistant gene was discovered in *Escherichia coli* in domestic swine in China^1^. This gene, the mobilized colistin resistance gene (*mcr-1*), is of critical concern because it confers resistance to colistin, a polymyxin antibiotic of last resort used in treating multi-drug resistant bacterial infections in humans^2^. Of particular concern for human health is the presence of the *mcr-1* gene in carbapenem-resistant Enterobacteriaceae, which could facilitate pandrug resistance^3^. Because the *mcr-1* gene is typically located on mobile genetic elements, it may be transferred among bacteria through horizontal gene transfer^4^; some bacterial infections could become difficult or impossible to treat if colistin resistance spreads to bacteria resistant to other antimicrobials^5^. Since 2015, *mcr-1* has been detected in at least 31 countries on six continents, including in the U.S.^5,6^, where it has been detected in human and domestic swine isolates from 21 states^7^.

Since initial discovery in domestic animals in China, *mcr-1* was found in fecal bacteria from European herring gulls (*Larus argentatus*) in Lithuania, kelp gulls (*Larus domnincanus*) in Argentina, and yellow-legged gulls (*Larus michahellis*) in Spain and Portugal^8–10^, which provides evidence that this gene may spread, in part, via environmental pathways. Gulls could play an important role in disseminating *mcr-1* among poultry, livestock, humans, and environmental sources given their wide-ranging movements, use of human waste sites and livestock feed, and propensity to spread bacterial pathogens^11,12^. For example, satellite-tracked glaucous-winged gulls (*Larus glaucescens*) made local movements up to 80 km during the breeding season, foraged at landfills during non-breeding periods, and migrated up to 3600 km in 63–75 days^13^. Gulls are also suspected to serve as reservoir (maintenance) hosts for antimicrobial resistant bacteria in general^14^ with evidence for dispersal of antimicrobial resistance genes across the landscape via local movements^15^. However, no studies to date have experimentally assessed whether gulls are readily colonized with bacteria harboring *mcr-1*, described patterns of shedding, or characterized how long fecally-shed bacteria may be detected in the environment.

In terms of disseminating microorganisms across the landscape, there are two host types of particular interest: maintenance hosts and bridge hosts^16^. Microorganisms persist in maintenance hosts even in the absence of transmission from other hosts while bridge hosts do not maintain microorganisms over extended periods but can transmit microorganisms from maintenance hosts to other populations^16^. In this study, we examined the research questions: *Are gulls readily colonized with mcr-1 positive E. coli and, if so, what is the magnitude and duration of shedding and of fecal contamination into the environment*? We also were interested in the question: *If gulls are readily colonized with mcr-1 positive E. coli, do they exhibit shedding patterns suggestive of maintenance or bridge hosts*^16^? To address these questions, we conducted an experiment where we inoculated ring-billed gulls (*Larus delawarensis*), a ubiquitous and wide-ranging species in North America^17^, with two strains of *mcr-1* positive *E. coli*. Within these strains, which were originally isolated from wild yellow-legged gulls in Spain (M175) and Portugal (POR1303), *mcr-1* exists in different genetic contexts. Specifically, *mcr-1* in POR1303 was chromosomally encoded and flanked by IS*Apl1* whereas *mcr-1* in M175 was located on an IncHI2 plasmid and was not flanked by IS*Apl1*^10^. Thus, the mobility of *mcr-1* genes harbored by these strains likely varies. We addressed the first research question by determining the magnitude and duration of fecal shedding over time for each of two m*cr-1* positive *E. coli* strains and by quantifying contamination from accumulated fecal material collected from the environment. We addressed the second research question by assessing whether uninoculated, co-housed individuals became colonized with *mcr-1* positive *E. coli* in a shared environment with inoculated gulls, and by measuring the deposition of the two strains into the environment via feces, which allowed us to assess whether ring-billed gulls exhibited patterns suggestive of maintenance or bridge hosts. We used curve fitting under an information-theoretic approach^18^ to provide evidence that gulls are more likely to be bridge or maintenance hosts under the assumption that shedding of *mcr-1* positive *E. coli* would peak and then decline to or near zero during the experimental period if gulls were bridge hosts or increase to some asymptote that remained at a relatively constant level if gulls were maintenance hosts.

## Results

We collected a total of 296 samples to assess bacterial colonization, shedding, transmission, and detection relative to the experimental inoculation of two groups of ring-billed gulls, each inoculated with a different strain of *mcr-1* positive *E. coli* and housed in separate rooms with contact controls. These samples included 240 swab samples (237 fecal samples and three cloacal swabs) from 16 individual gulls (11 inoculated birds and five contact controls), 32 water samples, and 24 aggregated fecal samples collected from designated sampling areas on the floors of the experimental rooms. Samples were collected every 2–3 days over a period of 33 days. We detected *E. coli* containing *mcr-1* in 8.8% of all samples (*n* = 26 of 296), as assessed by direct plating and following selective enrichment (see *Materials and Methods* section). Fecal samples from individuals weighed a mean of 0.26 g (95% CI = 0.23, 0.30) while the aggregated fecal samples from the floor weighed a mean of 2.98 g (95% CI = 2.24, 3.51).

### Shedding of *E. coli* containing *mcr-1* by individual gulls

**Figure 1:**
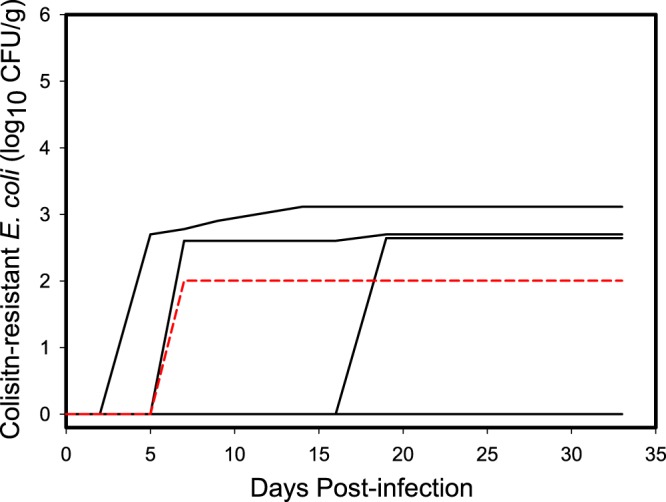
Cumulative shedding curves of *E. coli* strain M175 containing *mcr-1* by individual ring-billed gulls. Half of the six inoculated individuals shed similar cumulative amounts in their feces while the M175 strain was not detected in the feces of the other half. Dashed red line is the curve of the contact control bird that became colonized with *mcr-1* positive *E. coli* while housed with inoculated birds.

Prior to inoculation with the challenge strains, bacteria containing *mcr-1* was not detected in the feces of any of the gulls used in the experiment. After inoculation, we did not detect shedding of *E. coli* strain POR1303 in any of the swab samples (*n* = 120) collected from individual gulls inoculated with this bacterial strain. In contrast, we detected *E. coli* strain M175 in 6.7% (*n* = 8 of 120) of the swab samples collected from individual gulls inoculated with this strain. Half (3/6) of the individuals inoculated with the M175 strain shed this bacteria in their feces, although detection was sporadic. Over the 14 days where samples were collected during the 33-day period, the mean total shed was 10^2.57^ CFU/g of *mcr-1* positive *E. coli* (95% CI = 10^0^, 10^2.89^) across all individuals inoculated with strain M175 (Fig. 1) and 10^2.87^ (95% CI = 10^2.31^, 10^3.11^) across the three individuals that were positive for strain M175. Total cumulative shedding by individuals had a coefficient of variation (CV = SD/$\bar{x}$) of 136.6% indicating considerable individual heterogeneity, primarily due to detection of shedding in only half of the individuals. When considering only individuals that shed *mcr-1* positive *E. coli*, the coefficient of variation was reduced to 64.3%, suggesting more similar shedding patterns among colonized individuals (Fig. 1). In addition, one of the uninoculated contact control gulls became colonized with M175 *E. coli* and was observed shedding 10^2.00^ CFU/g on day 7 post inoculation (Fig. 1), indicating that this strain could be transmitted to conspecific birds.

### Shedding of *E. coli* containing *mcr-1* by gull flocks

**Figure 2:**
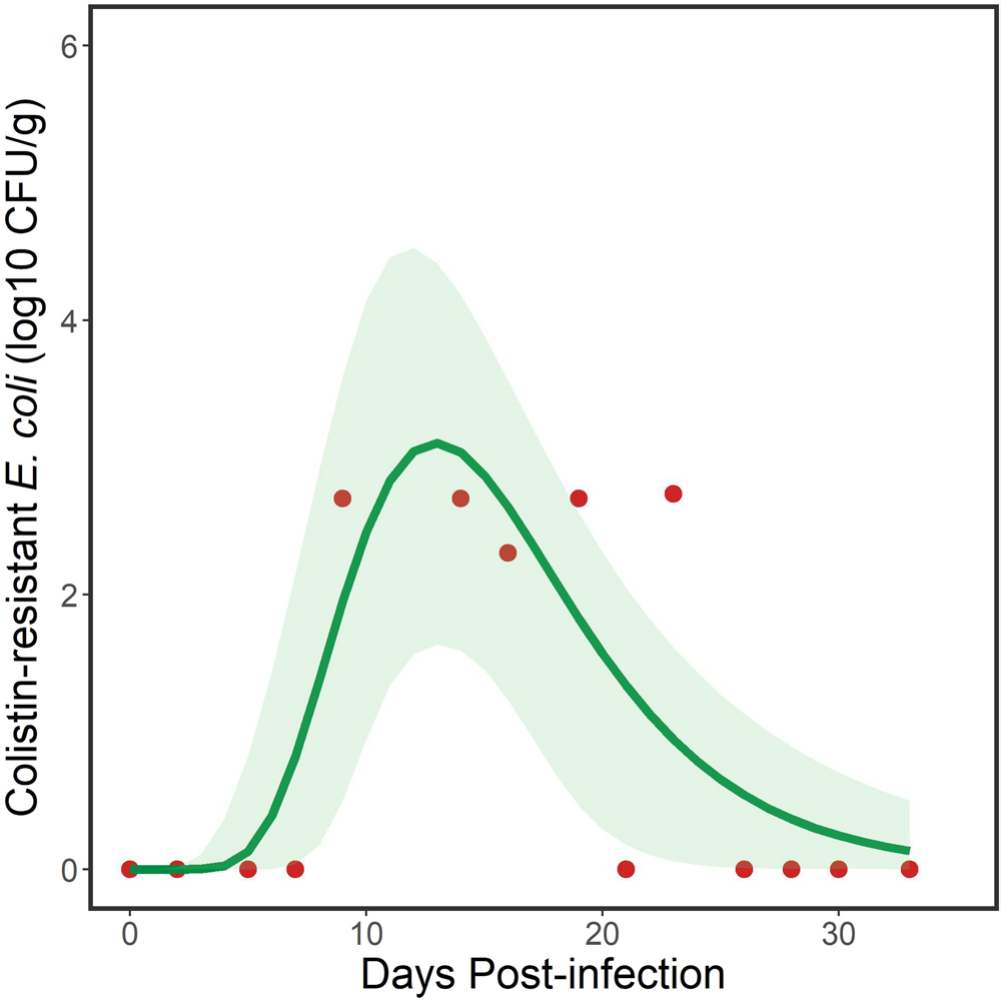
Fecal shedding of the M175 strain of *mcr-1* positive *E. coli* by a flock of ring-billed gulls. Data points (red dots) represent total daily amounts shed by individuals (*n* = 6) in the flock. Solid line represents modeled trend from a 3-parameter lognormal model and the green shaded area represents 95% confidence intervals. Note that detection of the target bacteria was sporadic.

Gulls tend to be highly gregarious, breed in colonies, and congregate in flocks during the nonbreeding season. For this reason, we statistically characterized bacterial shedding for each experimental group of gulls housed in the separate rooms, which we defined as a flock. Given that no birds shed strain POR1303, we only fit models to the challenge data for ringed-billed gulls inoculated with *E. coli* strain M175. Of the 27 models we fit to the flock shedding data for strain M175, a 3-parameter lognormal curve best explained the temporal pattern, based on minimum AICc (Table S1). This model took the form:

1 $$y=\frac{a}{x}\cdot {e}^{-0.5\cdot x{\left(\frac{{\rm{l}}{\rm{n}}(\frac{x}{{x}_{0}})}{b}\right)}^{2}}$$

where *y* = log_10_ CFU/g of *mcr-1* positive *E. coli*, *x* = DPI + 0.01, DPI is days post inoculation, *x*_0_ is the geometric mean (location parameter) that represents the peak *mcr-1* positive *E. coli* load shed by the flock, *b* is the geometric standard deviation (scale parameter) that defines the skewness and peakedness of the curve and *a* defined both the amplitude and area of distribution of the curve^19^ (Fig. 2). Parameter estimates for the fitted model (Fig. 2) are shown in Table 1. The model explained 65.5% of the variation in the data. The selection of this particular lognormal model suggested that gulls were not maintenance hosts but were acting as bridge hosts. That is, there was less support for relatively constant shedding of strain M175 by gulls over the duration of the study, which would be supported through selection of an intercepts-only or asymptotic model (Table S3) that suggested a maintenance host.

**Table 1:** Parameter estimates for 3-parameter lognormal models fit to fecal shedding by flocks of ring-billed gulls inoculated with two *mcr-1* positive strains of *E. coli* and environmental persistence of those strains.

Parameter	Estimate	Standard Error	95% Confidence Intervals
*Flock shedding of E. coli strain M175* (Fig. 2)			
*a*	43.35	8.45	24.74, 61.95
*b*	0.37	0.07	0.21, 0.53
*x*_0_	14.95	1.31	12.06, 17.84
*Environmental persistence of E. coli strain M175* (Fig. 3)			
*a*	44.74	8.04	26.84, 62.64
*b*	0.78	0.24	0.24, 1.32
*x*_0_	15.90	3.65	7.36, 23.62
*Environmental persistence of E. coli strain POR1303 (*Fig. 3)			
*a*	22.72	3.25	15.47, 29.97
*b*	0.51	0.29	−0.14, 1.17
*x*_0_	5.72	1.33	2.75, 8.69

Parameter *a* defines both the amplitude and area of distribution of the curve, *b* is the geometric standard deviation (scale parameter) that defines the skewness and peakedness of the curve, and *x*_0_ is the geometric mean (location parameter) that represents the peak *mcr-1* positive *E. coli* load.

The inverse of Eq. (1) yielded:

2 $$x={e}^{2.5673\pm \sqrt{-0.2761\cdot \mathrm{ln}(0.0231\cdot y)-0.7281}}$$

where the change in sign estimated the first day and last day, respectively, when a given log_10_ CFU/g of *mcr-1*-positive *E. coli* was detected. This equation was used to estimate the duration of shedding by the flock at the different concentrations of *mcr-1* positive *E. coli* that were detected, with 95% confidence intervals (Table 2). The model estimated that the inoculated flock of gulls shed at least 10^1^ CFU/g of *mcr-1* positive *E. coli* for 16.4 days, with shorter periods as the concentration increased (Table 2).

**Table 2:** Estimated number of days that gull flocks shed strains of *mcr-1* positive *E. coli* and that *E. coli* strains were detected in the environment at different bacterial concentrations.

Concentration	No. Days	95% Confidence Intervals
*Flock shedding of E. coli strain M175 (*Fig. 2)		
>10^1^	16.4	7.9, 26.7
>10^2^	10.3	3.7, 18.1
>10^3^	3.6	0.0, 11.3
*Environmental persistence of E. coli strain M175 (*Fig. 3)		
>10^1^	29.3	8.9, 50.3
>10^2^	18.4	6.2, 29.8
>10^3^	11.1	4.0, 20.0
*Environmental persistence of E. coli strain POR1303 (*Fig. 3)		
>10^1^	10.0	1.0, 21.3
>10^2^	7.3	1.0, 13.8
>10^3^	5.3	1.0, 9.9

Estimates were derived from the inverses of 3-parameter lognormal curves (see text).

### Environmental accumulation of *E. coli* containing *mcr-1* shed by gull flocks

We also examined the environmental accumulation of *mcr-1* positive *E. coli* from feces shed by the flock based on the aggregated fecal samples collected from designated areas on the floors of the experimental rooms. This differed from the previous analysis in that it characterized the fecal load of *mcr-1* positive *E. coli* in the environment that had been cumulatively deposited by the flock during the experimental period and which potentially allowed time for bacterial growth.

**Figure 3:**
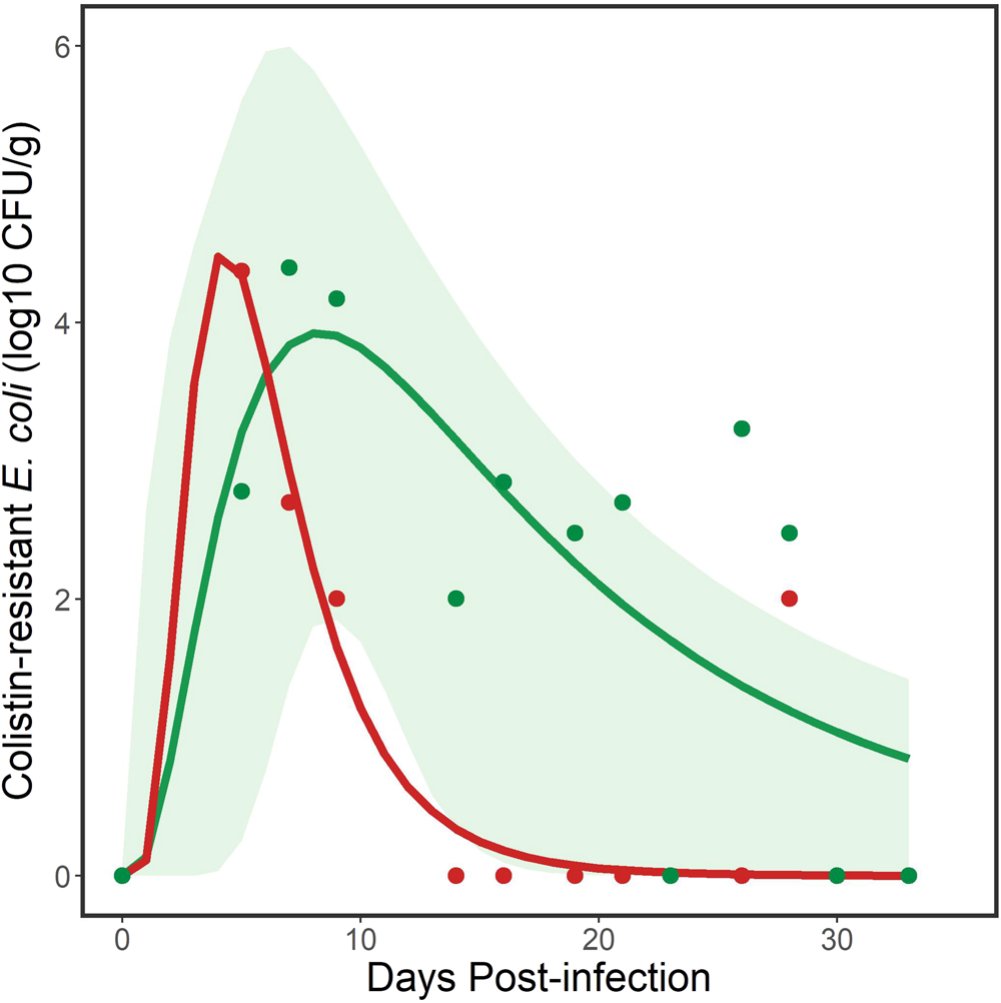
Environmental persistence of the M175 and POR1303 strains of *mcr-1* positive *E. coli* shed by a flock of ring-billed gulls. Data points (green dots for M175 and red dots for POR1303) are samples collected from the floor of the experimental room. Solid lines (green line for M175, red line for POR1303) represent modeled trends from 3-parameter lognormal models. Green shaded area represents 95% confidence intervals for modeled line for M175. Note that detection of the target bacteria was less sporadic than from the flock data (see Fig. 2). There was uncertainty in some of the data points for POR1303 (e.g., DPI 9 and 28), which were only positive following enrichment (see Materials and Methods). For this reason, 95% confidence intervals were not estimated for the modeled line for POR1303.

For the M175 strain, we used the same model set we fit to the flock data. A 3-parameter lognormal curve again best explained these data, based on minimum AICc (Table S2) with parameter estimates for the fitted model (Fig. 3) shown in Table 1. Based on AICc, a one compartment oral dose model was closely competitive with the 3-paramter lognormal curve. However, the resulting curves from the two models were nearly identical so we retained the 3-parameter lognormal curve for inferences. This model explained 59.3% of the variation in the data. The inverse of Eq. (1) with the floor sample parameter estimates yielded:

3 $$x={e}^{2.1260\pm \sqrt{-1.2284\cdot \mathrm{ln}(0.0224\cdot y)-2.9888}}$$

where the change in sign estimated the first day and last day, respectively, when a given log10 CFU/g of *mcr-1* positive *E. coli* was detected from aggregated fecal samples from the floor. These equations were used to estimate the persistence of the M175 strain of *mcr-1* positive *E. coli* cumulatively shed by the flock into the environment. At least 10^1^ CFU/g of *mcr-1* positive *E. coli* was estimated to persist in the environment used by the flock of gulls for 29.3 days (Table 2), which was longer than the estimated period derived using data for flocks and an equivalent bacterial concentration.

Although we did not detect the POR1303 bacterial strain in the feces of individual gulls, we did sporadically detect environmental contamination by this strain from gull feces collected on the floor of the experimental room for up to 28 days. Because data were sparse, we did not examine additional curves but fit a 3 parameter lognormal model for comparison with the other curves. This model took the same form as Eq. (1) with parameter estimates for the fitted model (Fig. 3) shown in Table 1. The model explained 82.6% of the variation in the data. The inverse of Eq. (1) with the different parameter estimates yielded:

4 $$x={e}^{1.4832\pm \sqrt{-0.5263\cdot \mathrm{ln}(0.04401\cdot y)-0.8417}}$$

where the change in sign estimated the first day and last day, respectively, when a given log10 CFU/g of *mcr-1* positive *E. coli* was detected from aggregated fecal samples from the floor. These equations were used to estimate the persistence of the POR1303 strain of *mcr-1* positive *E. coli* cumulatively shed by the flock into the environment. At least 10^1^ CFU/g of *mcr-1* positive *E. coli* persisted in the environment used by the flock of gulls for 10.0 days (Table 2), which was shorter than the estimated period of persistence for a similar concentration of the M175 strain. However, this reduced persistence was also a function of a smaller flock (*n* = 5) than the flock (*n* = 6) colonized with the M175 strain.

In both experiments, water samples yielded lower estimates of *mcr-1* positive *E. coli* concentrations as compared to feces collected from designated areas on the floor. Strain POR1303 was detected at 10^2.00^ CFU/g only once in water on day 7 post inoculation while the M175 strain was detected three times at 10^2.30^, 10^2.48^, and 10^2.00^ on days 5, 7 and 14 post inoculation, respectively.

### Effects of colonization of *E. coli* containing *mcr-1* on gulls

On the last day of the study (33 days post inoculation), all gulls were euthanized and then necropsied at the conclusion of the experiment. For each individual, we removed the proventriculus, gizzard, small intestine and large intestine and tested the contents (*n* = 48 from 16 individuals) for *mcr-1* positive *E. coli*. All necropsied samples tested negative for *mcr-1* positive *E. coli*. We detected no indication of disease and percent change in weights from the beginning of the study were greater for the uninoculated (control) gulls ($\bar{x}$ = −10.3%; 95% CI = −20.6, −2.8%; *n* = 4) than the inoculated gulls ($\bar{x}$ = −7.0%; 95% CI = −15.5, 1.5%; *n* = 12 including the colonized contact control). This indicated that colonization with the *mcr-1* positive *E. coli* strains used in this study did not cause weight loss or clinical disease.

## Discussion

In this study, we provided evidence that ring-billed gulls may be readily colonized with at least one strain of *mcr-1* positive *E. coli*, exhibit shedding patterns indicative of bridge hosts, shed bacteria in feces for an extended period (e.g., 16 days), which can be detected in the environment for even longer (e.g., 29 days), and are able to infect conspecifics occupying shared environments. Thus, we infer that ring-billed gulls have the potential to disseminate clinically important colistin resistance through environmental pathways.

Ring-billed gulls were readily colonized by one of two strains of *mcr-1* positive *E. coli* used as inoculum. This was evidenced by the detection of shedding in half of the inoculated birds as well as from one contact control. Reasons for apparent differences in infectivity of challenge strains are unclear, but could be related to differential host adaptation of strains used as inoculum, differences in detectability of the bacterial strains, heterogeneity among the immune response of birds used in this study, or other unknown factors. An additional possibility is that the M175 strain may be more detectable because the *mcr-1* gene was located on a plasmid, which could more easily spread to other *E. coli* in the gull’s gastrointestinal tract. As such, and because there was only a single replication of the experiment with each strain, we recommend caution in extrapolating these results to other strains of colistin-resistant bacteria, which may vary considerably in host responses.

In the absence of external selection pressures, the shedding pattern for ring-billed gulls challenged with *E. coli* strain M175 exhibited a relatively rapid initial increase in shedding to a peak followed by a slow decline in the amount shed^20^, which most closely fit our definition of a bridge host. That is, ring-billed gulls appear to be capable of shedding *E. coli* containing *mcr-1* for a sufficient period to transmit *mcr-1* to sympatric or conspecific birds and to contaminate occupied environments but *mcr-1* may not be maintained in the flock or population over extended periods without new inputs. However, the definitive status of a particular gull population as maintenance versus bridge hosts is dependent on population size^16^ so field studies are warranted to evaluate how our results would apply to natural populations. Generally, information on infectious doses to promote short-term growth of *mcr-1* positive *E. coli* in avian hosts is lacking. However, infectious doses of pathogenic *E. coli* in humans range from 10^1^ to 10^3^ CFU^21,22^. If 10^2^ CFU is considered a reasonable dose to infect a vertebrate host with *mcr-1* positive *E. coli*, then based on our estimates, small flocks of gulls could shed infectious doses of strain M175 for approximately 10 days, which persist in the environment for up to approximately 18 days. However, the discrete distinction between bridge and maintenance host may be blurred in gulls because of their flocking behavior coupled with selection pressures in a contaminated real-world environment that could act to maintain colonization of *mcr-1* positive *E. coli* in flocks of gulls that can number in hundreds and thousands^23^.

**Figure 4:**
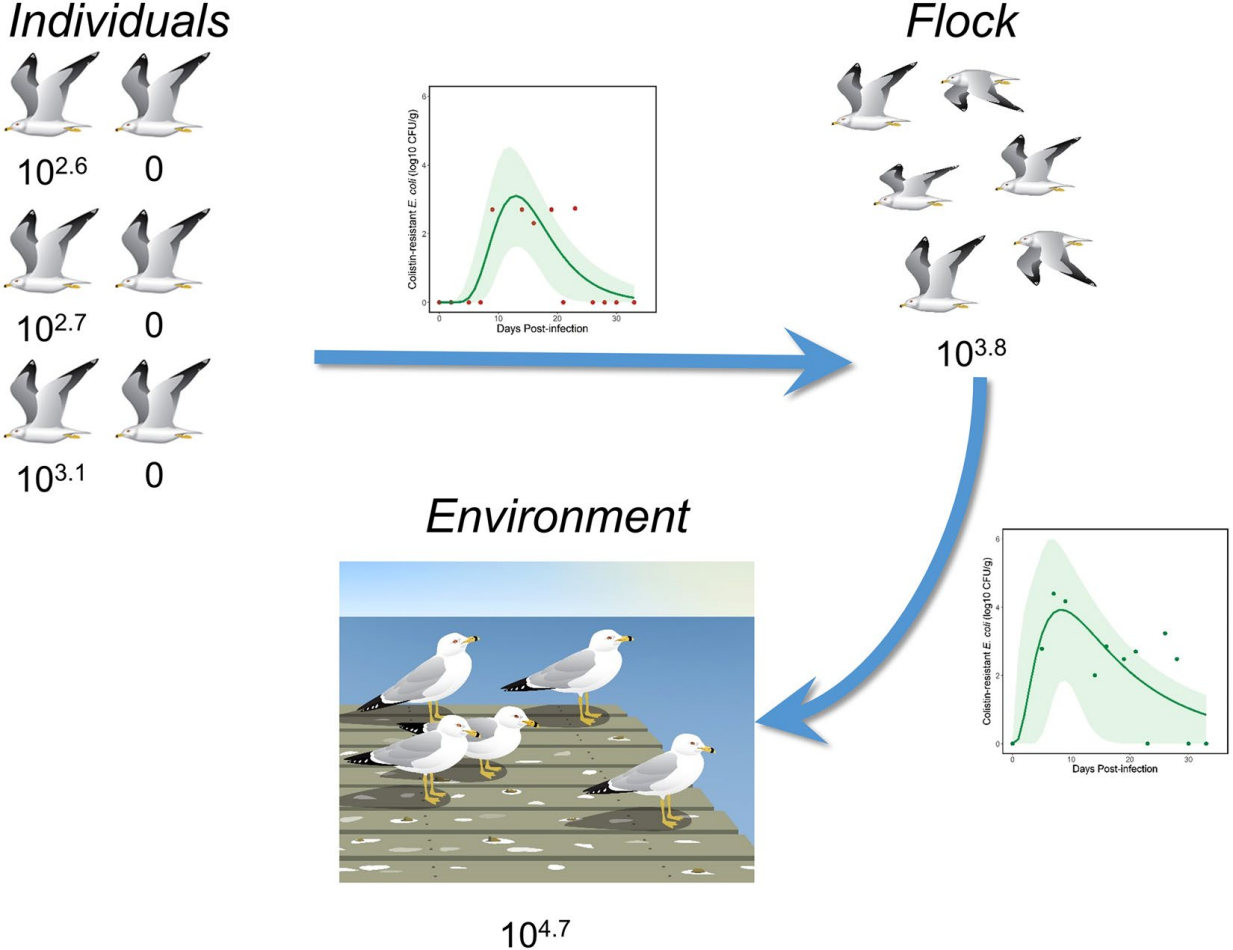
Differential estimates of deposition (in CFU/g) of strain M175 *mcr-1* positive *E. coli* when assessed at different scales. Arrows represent progression from smaller (individuals) to larger scales (flocks and the environment). Estimates represent total accumulations within the flock and environment categories based on the area under the modeled growth curves (see Figs. 2 and 3). Individual estimates are shown for comparison but represent amounts measured every 2–3 days and do not accurately represent total amounts shed by individuals. Images were drawn by Vanessa Sorensen of Sorensen Designs.

In terms of contamination risk, the estimated amount of *E. coli* containing *mcr-1* bacteria in environments being used by gulls appeared to increase by an order of magnitude when going from fecal output by individuals comprising the flock to accumulated feces deposited in the environment (Fig. 4). This can be explained by detectability issues, bacterial growth in the environment, or a combination of both. Clearly, there are detectability issues as exemplified by lack of detection of strain POR1303 in individual gulls despite detection in water and on the floor in our closed system. In this case, sample volume may have affected detectability. Fecal amounts collected directly from the gulls were low relative to those collected from the floors of experimental rooms and we suspect that proportionally smaller fecal samples from individual birds contributed to the lack of detection of bacteria. In addition, *E. coli* has the potential to grow in the environment; for example, beach sand has been suggested as an environment in which *E. coli* can replicate^24^. It is plausible that bacterial growth in fecal samples collected from the floor of experimental rooms, coupled with their larger volumes may have contributed to greater detectability. Regardless, we provide evidence that gulls ultimately contaminated the environment with substantial amounts of viable *mcr-1* positive *E. coli*, which was transmitted to at least one contact control in the case of strain M175.

We found large differences in the two strains of *mcr-1* positive *E. coli* we tested in this experiment. The M175 strain was considered to be more prone to horizontal gene transfer (plasmid-encoded) of *mcr-1* gene while the POR1303 likely had a more genetically stable *mcr-1* gene (chromosomally encoded)^10^. We are unsure if this difference affected the detectability or shedding characteristics of the two strains or whether there were other responsible genetic differences. For example, chromosomally encoded carbapenemase (*bla*_KPC_) genes in *Klebsiella pneumoniae* were more difficult to phenotypically detect than those which were plasmid encoded^25^. From a surveillance perspective, minimal detection of *mcr-1* positive *E. coli* in feces collected from individual birds, despite detection in fecal material accumulated in occupied environments, suggests that surveillance for clinically relevant antibiotic resistance targets should be conducted through environmental sampling of extensively used areas rather than by sampling individual birds.

**Figure 5:**
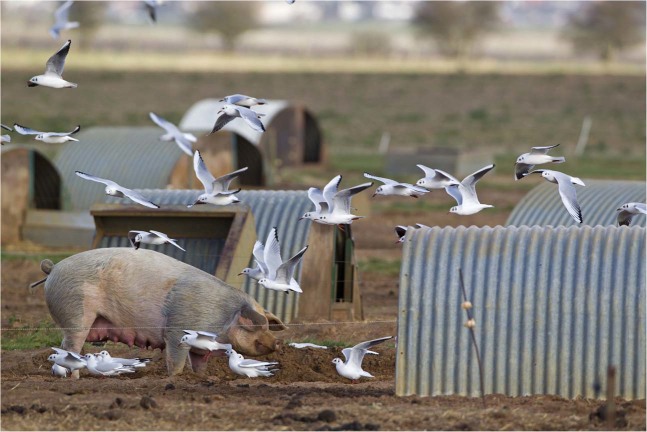
Example of gulls interacting with agricultural livestock operations. Photograph is of a flock of black-headed gulls (*Larus ridibundus*) feeding at an outdoor pig unit in January, Suffolk, England, which illustrates one mechanism by which *mcr-1* positive *E. coli* could be disseminated between gulls and domestic animals consumed by the humans. Photograph is **©**age footstock America Inc.

We propose that gulls serve as useful sentinels for *mcr-1* and other clinically relevant forms of colistin resistance in the environment because of the mobility of gulls, their ability to disseminate both pathogenic and antimicrobial resistant bacteria throughout agricultural landscapes^26^ and environments used by people^12,27,28^, the apparent asymptotic colonization of experimentally challenged birds with *E. coli* harboring colistin resistance, and the evidence for extensive environmental contamination by gulls via fecal shedding. Furthermore, we propose that gulls play a role in environmental pathways through which *mcr-1* positive *E. coli* could be disseminated to humans or domestic animals through fecal contamination of public areas by gulls, e.g.^12^, or agricultural operations^26^ (Fig. 5).

## Methods

### Experimental design

In designing this experiment, we developed hypotheses about both host characteristics and bacterial strain characteristics. Research hypotheses concerning host characteristics included (1) gulls are maintenance hosts^16^, defined in this study as maintaining a constant rate of fecal shedding of *E. coli* containing *mcr-1* at some infectious dose for 30 days (i.e., capable of infecting uninoculated individuals sharing the same resources), and (2) gulls are bridge hosts^16^, defined in this study as maintaining a declining rate of fecal shedding of *E. coli* containing *mcr-1* at a colonization dose for <30 days). We assumed that transmission of bacteria between individuals would be through fecal shedding and that transmission probability was thus proportional to bacterial amounts shed in feces. There were no data to support how long a period is needed to differentiate maintenance from bridge hosts so we *a priori* assumed that 30 days would be sufficient to differentiate between the two host types.

We used ring-billed gulls (*Larus delawarensis*) as our study subject because they are found across much of North America, are often associated with anthropogenically influenced habitats including landfills and agricultural fields, and had the potential to move *mcr-1* positive *E. coli* through both migratory and local movements^17^. We captured gulls from a landfill near Fort Collins, Larimer County, Colorado. We experimentally inoculated two groups of gulls, each with a different strain of *E. coli* harboring the *mcr-1* gene. The first group was inoculated with *E. coli* strain POR1303 and the second group with *E. coli* strain M175, both of which were isolated from yellow-legged gulls (*Larus micahellis*) in Portugal and Spain, respectively, in 2009. The two strains differed in that POR1303 had two complete copies of IS*Apl1* flanking the chromosomally encoded *mcr-1* gene while the M175 strain lacked any IS*Apl1* elements and *mcr-1* was located on a plasmid^10^. Within each group of gulls, we used 8 gulls. Each group was housed in a separate Biosafety level 2 room designed for wildlife experimental infections. In one room, we inoculated 5 gulls with the POR1303 strain and included 3 uninoculated gulls as contact controls. In the second room, we inoculated 6 gulls with strain M175 and included 2 uninoculated gulls as contact controls. Bacterial strains were randomly assigned to the experimental rooms and then gulls were assigned to rooms and inoculation status using a stratified random assignment where stratification was by sex. This allowed for approximately equal representation of sexes in the two groups while randomly assigning gulls to strains and inoculation status.

### Experimental procedures

Prior to inoculating gulls with the bacterial strains, we collected fecal samples from each individual 5, 2 and 0 days prior to inoculation to ensure gulls were not colonized with bacteria containing the *mcr-1* gene. Gulls assigned to treatments were inoculated with approximately 1 × 10^6^ cfu/mL of the bacterial strains delivered in 2 mL of sterile nonfat milk, which was administered orally. The inoculum was prepared by growing the *mcr-1 E. coli* strains overnight in tryptic soy broth (TSB) at 37 °C, followed by dilution of these cultures to the target inoculum concentration in sterile nonfat milk. Inoculums were enumerated as described in the section below. This dose was similar to infectious doses for enteroinvasive and enteropathogenic *E. coli*^29,30^. Gulls were allowed to move freely about the experimental rooms, although their wings were clipped to prevent flight. Every 2–3 days, all individuals were captured, placed into individual cages with clean butcher paper and allowed to defecate. Once individuals defecated within the cage, feces were collected using a sterile spatula and placed into a pre-weighed, sterile 50 mL centrifuge tube from which fecal weights were determined. If individuals did not defecate within 1 hr, fecal material was extracted using a cloacal swab. In addition, a 5 mL sample was taken using a sterile pipette from the ~4 L water bowl placed in each room; water in this bowl was changed every week over the course of the experiment. Because gulls were captured at a landfill and were acclimated to a nontraditional diet, they were fed *ad libitum* with canned dog food and pre-cooked hot dogs, which they preferred over other food items. As a measure of environmental contamination, we also collected a conglomerate of fecal samples from four 61 × 92 cm sheets of butcher paper taped to the floor in areas where the gulls tended to loaf. Samples from the paper were collected every 2–3 days and the paper was replaced after each sampling. All samples were placed on ice following collection, and processed in the laboratory within 2 hr. After inoculation of gulls, samples were collected over a 33-day period after which all gulls were euthanized by injecting 0.5 mL of B-Euthosol in 0.5 mL of sterile water into the brachial vein. All carcasses were necropsied.

All animal work described here was carried out in strict accordance with the Animal Welfare Act. The study protocol (QA-2882) was approved and overseen by the National Wildlife Research Center Institutional Animal Care and Use Committee and Attending Veterinarian. Gulls were captured and maintained in captivity under U.S. Fish and Wildlife Service Scientific Collecting permit number MB19065-3 and Colorado Parks and Wildlife Scientific Collection License number 18TRb2433.

### Laboratory analysis of fecal samples and bacterial isolates

Gull fecal samples were suspended and homogenized in TSB in preparation for microbiological analyses. Given the variability and relatively low fecal mass of many of these samples (weight of fecal samples from individual birds ranged from 0.01 g to 2.5 g) and volumetric requirements for subsequent analyses, samples were diluted between 1:10–1:140 in TSB. From this suspension, 500 µL aliquots (each) were taken for direct enumeration of target bacteria by plating and for enrichments (selective and non-selective). For direct enumeration, samples were plated on CHROMagar COL-APSE (CHROMagar, Paris, France) supplemented with 1 µg/mL of ciprofloxacin (Millipore-Sigma, St. Louis, MO, USA) and incubated at 37 °C for 18 hr. Only colonies displaying a typical *E. coli* morphology on this media were counted. Antimicrobial susceptibility testing determined both *E. coli* strains containing *mcr-1* used in this study (POR1303 and M175) were ciprofloxacin-resistant, and addition of this antibiotic adequately suppressed growth of microbiota found in gull feces otherwise able to grow on CHROMagar COL-APSE, facilitating reliable enumeration. 500 µL aliquots of the fecal slurry were enriched in 4.5 mL of TSB and TSB supplemented with 1 µg/mL colistin (Millipore-Sigma) for 18 hr at 37 °C to better establish presence/absence of the target bacteria as compared to direct plating. Following enrichment, a 50 µL aliquot of each sample was inoculated to CHROMagar COL-APSE with ciprofloxacin and incubated at 37 °C for 18 hr. The sample was considered positive by enrichment if colonies typical of *mcr-1* positive *E. coli* were confirmed.

Inoculum and select bacterial colonies from both direct plating and from enrichments were confirmed as *E. coli* by MALDI Biotyping in accordance with standard procedures^31^. Additionally, the presence of *mcr-1* was confirmed in all these isolates, following boil prep, by RT-PCR using the procedure developed by Irrgang *et al*.^32^. For all isolates analyzed, those displaying typical morphologies on CHROMagar COL-APSE with ciprofloxacin were always confirmed as *mcr-1* positive *E. coli*.

### Statistical analysis

We analyzed individual shedding characteristics as cumulative curves over the 33-day post-inoculation period. We used coefficient of variation (CV = SD/mean) of the cumulative amount shed as an estimate of individual heterogeneity. We fit curves from different models to our data because (1) we sampled every 2–3 days and models allowed us to extrapolate between data points, and (2) models allowed us to generalize temporal patterns and estimate key parameters of interest with measures of precision. To examine temporal patterns in shedding by flocks and environmental persistence and to test hypotheses concerning host type, we fit a means (intercept only) model and 26 non-linear models (Table S3) that could be either expressed as a peak, asymptote or both to all the data (both positive and negative). For the flock data, we used the daily amount of *mcr-1* positive *E. coli* shed by all the individuals in the flock, We used a bias-corrected version of Akaike’s Information Criterion (AICc)^33^ in an information-theoretic approach^18^ to select the best model from this set of models. We used packages *nlstools*^34^ and the nlsLM function in package *minpack*^35^ in the R statistical software system^36^ to fit nonlinear models and the GENMOD procedure^37^ in SAS to fit polynomial and means models to the data using maximum likelihood. Selection of the means or asymptotic models using the information-theoretic approach would have supported the hypothesis that gulls were maintenance hosts while selection of any of the peaked nonlinear models would have supported the hypothesis that gulls were bridge hosts per our definitions for this study. The proportion of variation in the data explained by the models was estimated using R code in^38^ and 95% confidence intervals around estimated curves was estimated using the predictNLS function in the *propagate* R package^39^.

To estimate the number of days that *mcr-1* positive *E. coli* was detected at certain levels (e.g., >10^1^, >10^2^ CFU/g, etc.), we took the inverse of the function for the selected model. We then solved the inverse function using the point, lower 95% confidence interval and upper 95% confidence interval parameter estimates for the selected model. This provided us with a set of equations to estimate the DPI when a certain level of *mcr-1* positive *E. coli* was first and last detected, and, ultimately, the number of days in between, along with 95% confidence intervals for those estimates. These estimates provided a measure of shedding or persistence duration for given levels of *mcr-1* positive *E. coli*.

## Supplementary information

Supplementary information.

## Data Availability

All data are archived at the Information Services Unit of the USDA National Wildlife Research Center, Fort Collins, CO.
