# Supplementary material for: Gulls as Sources of Environmental Contamination by Colistin-resistant Bacteria
Source: Sci Rep. 2020 Mar 10;10:4408. doi: 10.1038/s41598-020-61318-2 (PMC7064522; doi:10.1038/s41598-020-61318-2)
Supplement: Supplementary file 1 — Supplementary information. [file 41598_2020_61318_MOESM1_ESM.docx]

SUPPLEMENTARY MATERIALS

Gulls as Sources of Environmental Contamination by Colistin-resistant Bacteria

Alan B. Franklin, Andrew M. Ramey, Kevin T. Bentler, Nicole L. Barrett, Loredana M. McCurdy, Christina A. Ahlstrom, Jonas Bonnedahl, Susan A. Shriner, and Jeffrey C. Chandler

Corresponding author: Alan B. Franklin ([alan.b.franklin@usda.gov](mailto:alan.b.franklin@usda.gov))

This supplement comprises:

Supplementary Tables S1-S3

**Table S1. Model selection results for 27 growth curves fit to daily shedding (log_10_ CFU/g) by a flock of ring-billed gulls experimentally inoculated with the M175 strain of *mcr-1* positive *E. coli*.** Bolded model indicates selected model based on minimum AICc.

| **Curve** | **-2lnL^a^** | **K^b^** | **AICc^c^** | **∆AICc^d^** | **Akaike Weight** |
| --- | --- | --- | --- | --- | --- |
| Means (Intercept Only) | 48.048 | 2 | 53.139 | 9.320 | 0.004 |
| Gaussian 3 Parameter | 33.164 | 4 | 45.608 | 1.790 | 0.190 |
| Gaussian 4 Parameter | 32.951 | 5 | 50.451 | 6.632 | 0.017 |
| Modified Gaussian 4 Parameter | No convergence | | |  |  |
| Modified Gaussian 5 Parameter | No convergence | | |  |  |
| Generalized Gaussian | No convergence | | |  |  |
| Lorenentzian 3 Parameter | 35.190 | 4 | 47.634 | 3.815 | 0.069 |
| Lorenentzian 4 Parameter | No convergence | | |  |  |
| Pseudo-Voigt 4 Parameter | 32.648 | 5 | 50.148 | 6.329 | 0.020 |
| Pseudo-Voigt 5 Parameter | 32.511 | 6 | 56.511 | 12.692 | 0.001 |
| **Lognormal 3 Parameter** | **31.374** | **4** | **43.819** | **0.000** | **0.466** |
| Lognormal 4 Parameter | No convergence | | |  |  |
| Weibull 4 Parameter | 32.594 | 5 | 50.094 | 6.275 | 0.020 |
| Weibull 5 Parameter | 41.994 | 6 | 65.994 | 22.176 | 0.000 |
| Polynomial 2nd Order | 37.170 | 4 | 49.615 | 5.796 | 0.026 |
| Polynomial 2nd Order without intercept | 37.735 | 3 | 46.135 | 2.316 | 0.146 |
| Polynomial 3rd Order | 37.734 | 4 | 50.178 | 6.360 | 0.019 |
| Polynomial 3rd Order without intercept | No convergence | | |  |  |
| Polynomial 4th Order | 32.766 | 6 | 56.766 | 12.948 | 0.001 |
| Polynomial 4th Order without intercept | 32.777 | 5 | 50.277 | 6.459 | 0.018 |
| Polynomial 5th Order | 31.989 | 7 | 64.656 | 20.837 | 0.000 |
| Polynomial 5th Order without intercept | 32.027 | 6 | 56.027 | 12.208 | 0.001 |
| Polynomial 6th Order | 31.962 | 8 | 76.762 | 32.944 | 0.000 |
| Polynomial 6th Order without intercept | 32.016 | 7 | 64.683 | 20.864 | 0.000 |
| One Compartment Oral Dose | No convergence | | |  |  |
| Biexponential 4 Parameter | 41.025 | 5 | 58.525 | 14.707 | 0.000 |
| Biexponential 5 Parameter | No convergence | | |  |  |
| ^a^-2 logLikelihood  ^b^number of parameters  ^c^bias-corrected Akaikes Information Criterion  ^d^Difference (in AICc units) from model with lowest AICc value | | | | | |

**Table S2. Model selection results for 27 growth curves fit to environmental persistence (log_10_ CFU/g) of the M175 strain of *mcr-1* positive *E. coli* shed by experimentally inoculated ring-billed gulls.** Bolded model indicates selected model based on minimum AICc.

| **Curve** | **-2lnL^a^** | **K^b^** | **AICc^c^** | **∆AICc^d^** | **Akaike Weight** |
| --- | --- | --- | --- | --- | --- |
| Means (Intercept Only) | 54.059 | 2 | 59.150 | 10.568 | 0.002 |
| Gaussian 3 Parameter | 41.003 | 4 | 53.448 | 4.866 | 0.028 |
| Gaussian 4 Parameter | 40.350 | 5 | 57.850 | 9.269 | 0.003 |
| Modified Gaussian 4 Parameter | 40.088 | 5 | 57.588 | 9.007 | 0.004 |
| Modified Gaussian 5 Parameter | 40.052 | 6 | 64.052 | 15.471 | 0.000 |
| Generalized Gaussian | No convergence | | |  |  |
| Lorenentzian 3 Parameter | 40.646 | 4 | 53.091 | 4.509 | 0.034 |
| Lorenentzian 4 Parameter | No convergence | | |  |  |
| Pseudo-Voigt 4 Parameter | 39.622 | 5 | 57.122 | 8.540 | 0.004 |
| Pseudo-Voigt 5 Parameter | 38.303 | 6 | 62.303 | 13.722 | 0.000 |
| **Lognormal 3 Parameter** | **36.137** | **4** | **48.581** | **0.000** | **0.322** |
| Lognormal 4 Parameter | 36.136 | 5 | 53.636 | 5.054 | 0.026 |
| Weibull 4 Parameter | 43.807 | 5 | 61.307 | 12.726 | 0.001 |
| Weibull 5 Parameter | 43.943 | 6 | 67.943 | 19.362 | 0.000 |
| Polynomial 2nd Order | 40.885 | 4 | 53.329 | 4.748 | 0.030 |
| Polynomial 2nd Order without intercept | 42.903 | **3** | 51.303 | 2.722 | 0.082 |
| Polynomial 3rd Order | 38.622 | **5** | 56.122 | 7.541 | 0.007 |
| Polynomial 3rd Order without intercept | 38.896 | 4 | 51.340 | 2.759 | 0.081 |
| Polynomial 4th Order | 34.039 | 6 | 58.039 | 9.458 | 0.003 |
| Polynomial 4th Order without intercept | 34.050 | 5 | 51.550 | 2.969 | 0.073 |
| Polynomial 5th Order | 34.035 | 7 | 66.702 | 18.120 | 0.000 |
| Polynomial 5th Order without intercept | 34.044 | 6 | 58.044 | 9.463 | 0.003 |
| Polynomial 6th Order | 32.628 | 8 | 77.428 | 28.846 | 0.000 |
| Polynomial 6th Order without intercept | 32.628 | 7 | 65.294 | 16.713 | 0.000 |
| One Compartment Oral Dose | 36.455 | 4 | 48.899 | 0.318 | 0.274 |
| Biexponential 4 Parameter | 36.453 | 5 | 53.953 | 5.372 | 0.022 |
| Biexponential 5 Parameter | 36.077 | 6 | 60.077 | 11.496 | 0.001 |
| ^a^-2 logLikelihood  ^b^number of parameters  ^c^bias-corrected Akaikes Information Criterion  ^d^Difference (in AICc units) from model with lowest AICc value |  |  |  |  |  |

**Table S3. Candidate models fit to flock shedding and environmental persistence data for *mcr-1* positive *E.coli* shed by experimentally inoculated ring-billed gulls.** In all cases the response variable was (log_10_CFU/g mcr-1 positive *E. coli*+1) and the explanatory variable was days post inoculation (DPI).

| **Curve** | **Equation (in R code format)** | **Curve Form** |
| --- | --- | --- |
| Means (Intercept Only) | b0 | Asymptotic |
| Gaussian 3 Parameter | a*exp(-0.5*((DPI-x0)/b)^2) | Peak |
| Gaussian 4 Parameter | y0+a*exp(-0.5*((DPI-x0)/b)^2) | Peak |
| Modified Gaussian 4 Parameter | a*exp(-0.5*abs((DPI-x0)/b)^c) | Peak |
| Modified Gaussian 5 Parameter | y0+a*exp(-0.5*abs((DPI-x0)/b)^c) | Peak |
| Generalized Gaussian | (b/(2*a*gamma(1/b)))*exp(-(abs(DPI-x0)/a)^b) | Peak |
| Lorentzian 3 Parameter | a/(1+((DPI-x0)/b)^2) | Peak |
| Lorentzian 4 Parameter | y0+(a/(1+(DPI-x0)/b)^2) | Peak |
| Pseudo-Voigt 4 Parameter | a*(c*(1/(1+((DPI-x0)/b)^2))+(1-c)*exp(-0.5*((DPI-x0)/b)^2)) | Peak, Asymptotic |
| Pseudo-Voigt 5 Parameter | y0+(a*(c*(1/(1+((DPI-x0)/b)^2))+(1-c)*exp(-0.5*((DPI-x0)/b)^2))) | Peak, Asymptotic |
| Lognormal 3 Parameter | a*exp(-0.5*(log((DPI+.1)/x0)/b)^2)/(DPI+.1) | Peak, Asymptotic |
| Lognormal 4 Parameter | y0+(a*exp(-0.5*(log((DPI+.1)/x0)/b)^2)/(DPI+0.1)) | Peak, Asymptotic |
| Weibull 4 Parameter | a*((c-1)/c)^((1-c)/c)*(abs(((DPI+0.1)-x0)/b+((c-1)/c)^(1/c))^(c-1))*exp(-abs(((DPI+0.1)-x0)/b+((c-1)/c)^(1/c))^c+(c-1)/c) | Peak, Asymptotic |
| Weibull 5 Parameter | y0+(a*((c-1)/c)^((1-c)/c)*(abs(((DPI+0.1)-x0)/b+((c-1)/c)^(1/c))^(c-1))*exp(-abs(((DPI+0.1)-x0)/b+((c-1)/c)^(1/c))^c+(c-1)/c)) | Peak, Asymptotic |
| Polynomial 2nd Order | b0+b1*DPI+b2*DPI^2 | Peak, Asymptotic |
| Polynomial 2nd Order without intercept | b1*DPI+b2*DPI^2 | Peak, Asymptotic |
| Polynomial 3rd Order | b0+b1*DPI+b2*DPI^2+b3*DPI^3 | Peak, Asymptotic |
| Polynomial 3rd Order without intercept | b1*DPI+b2*DPI^2+b3*DPI^3 | Peak, Asymptotic |
| Polynomial 4th Order | b0+b1*DPI+b2*DPI^2+b3*DPI^3+b4*DPI^4 | Peak, Asymptotic |
| Polynomial 4th Order without intercept | b1*DPI+b2*DPI^2+b3*DPI^3+b4*DPI^4 | Peak, Asymptotic |
| Polynomial 5th Order | b0+b1*DPI+b2*DPI^2+b3*DPI^3+b4*DPI^4+b5*DPI^5 | Peak, Asymptotic |
| Polynomial 5th Order without intercept | b1*DPI+b2*DPI^2+b3*DPI^3+b4*DPI^4+b5*DPI^5 | Peak, Asymptotic |
| Polynomial 6th Order | b0+b1*DPI+b2*DPI^2+b3*DPI^3+b4*DPI^4+b5*DPI^5+b6*DPI^6 | Peak, Asymptotic |
| Polynomial 6th Order without intercept | b1*DPI+b2*DPI^2+b3*DPI^3+b4*DPI^4+b5*DPI^5+b6*DPI^6 | Peak, Asymptotic |
| One Compartment Oral Dose | (a*b*c)/(c-b))*(exp(-b*DPI)-exp(-c*DPI)) | Peak |
| Biexponential 4 Parameter | a*exp(-b*DPI)+c*exp(-d*DPI) | Peak, Asymptotic |
| Biexponential 5 Parameter | y0+a*exp(-b*DPI)+c*exp(-d*DPI) | Peak, Asymptotic |
